# Al_2_O_3_ Dot and Antidot Array Synthesis in Hexagonally Packed Poly(styrene-*block*-methyl methacrylate) Nanometer-Thick Films for Nanostructure Fabrication

**DOI:** 10.1021/acsanm.2c02013

**Published:** 2022-07-05

**Authors:** Gabriele Seguini, Alessia Motta, Marco Bigatti, Federica E. Caligiore, Guido Rademaker, Ahmed Gharbi, Raluca Tiron, Graziella Tallarida, Michele Perego, Elena Cianci

**Affiliations:** † IMM-CNR, Unit of Agrate Brianza, Via C. Olivetti 2, Agrate Brianza I-20864, Italy; ‡ Univ. Grenoble Alpes, CEA, Leti, Grenoble F-38000, France

**Keywords:** BCP, SIS, VPI, PMMA, TMA, Al_2_O_3_, dot, antidots

## Abstract

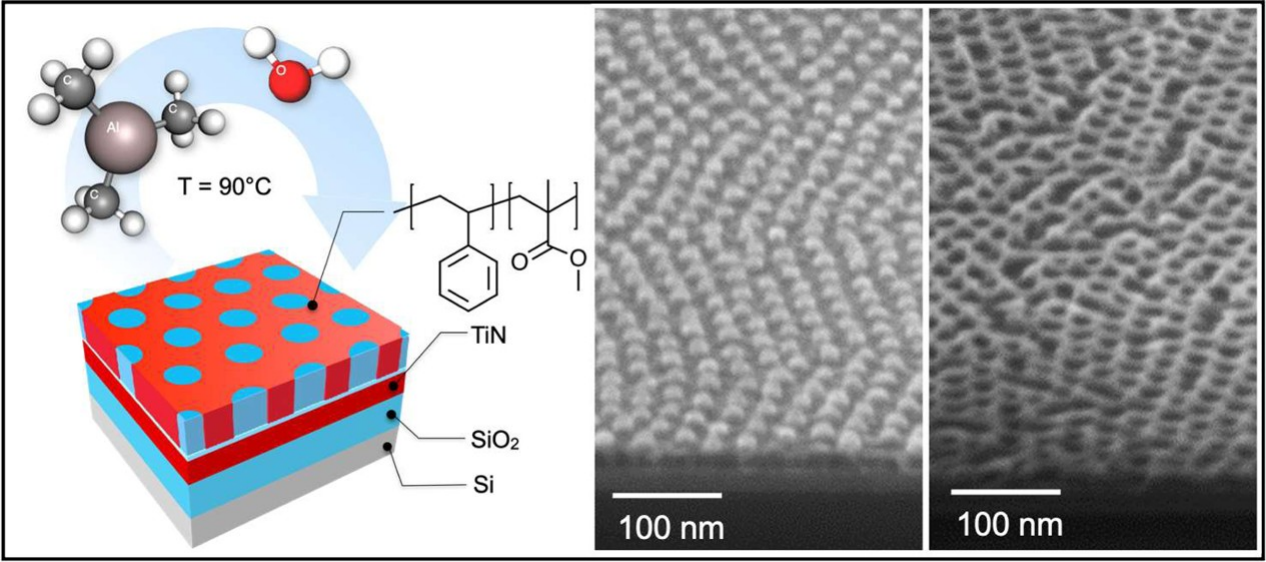

Nanostructured organic templates originating from self-assembled block copolymers (BCPs) can be converted into inorganic nanostructures by sequential infiltration synthesis (SIS). This capability is particularly relevant within the framework of advanced lithographic applications because of the exploitation of the BCP-based nanostructures as hard masks. In this work, Al_2_O_3_ dot and antidot arrays were synthesized by sequential infiltration of trimethylaluminum and water precursors into perpendicularly oriented cylinder-forming poly(styrene-*block*-methyl methacrylate) (PS-*b*-PMMA) BCP thin films. The mechanism governing the effective incorporation of Al_2_O_3_ into the PMMA component of the BCP thin films was investigated evaluating the evolution of the lateral and vertical dimensions of Al_2_O_3_ dot and antidot arrays as a function of the SIS cycle number. The not-reactive PS component and the PS/PMMA interface in self-assembled PS-*b*-PMMA thin films result in additional paths for diffusion and supplementary surfaces for sorption of precursor molecules, respectively. Thus, the mass uptake of Al_2_O_3_ into the PMMA block of self-assembled PS-*b*-PMMA thin films is higher than that in pure PMMA thin films.

## Introduction

Atomic layer deposition (ALD) on polymers^1−3^ initiated a three-dimensional (3D) growth process of inorganic materials into polymeric films known as sequential infiltration synthesis (SIS) or vapor phase infiltration (VPI).^4−7^ Several studies have been published to investigate the fundamental physical chemistry of the SIS/VPI process.^8−11^ The understanding of the basic mechanisms governing the process allowed expanding the list of materials that can be grown using this technique. In particular, the growth of several oxides, like Al_2_O_3_, TiO_2_,^12^ ZnO,^12−15^ WO_*x*_,^16^ VO_*x*_,^12^ In_2_O_3_,^17,18^ Ga_2_O_3_,^18^ and SnO_2_,^19^ has been already reported in the literature for different applications, such as high-resolution hard masks,^20−25^ nanoparticle coatings and decoration,^8,26,27^ superhydrophobic coatings,^28^ optical materials and antireflection coatings,^29,30^ enhancer of the contrast and scattering of nanostructures,^24,31,32^ 3D superlattices,^33^ oil sorbents,^34^ UV and thermal protection,^14^ tuning of mechanical propertries,^35^ sensing applications,^15^ membranes,^36−38^ elastic energy-storage structures,^39^ electrical devices,^13,17,40,41^ and resistive switching devices.^42^ In addition, SIS can be also exploited as a postlithography technique to improve the extreme ultraviolet patterning process.^43^ Waldman et al. provided a systematic and comprehensive survey about current SIS results in the literature.^5^

When SIS is performed into self-assembled block copolymers (BCPs), the selective binding of precursors to one domain of BCPs permits fabricating inorganic nanostructures or hard masks for lithography.^44,45^ In BCPs, the repulsive interaction between the covalently bonded component blocks leads to microphase separation into periodic nanostructures (e.g., spheres, cylinders, lamellae, and gyroids) depending on the volume ratio of the two blocks.^46^ BCP self-assembly and the SIS growth process are two separate steps. In a self-assembled BCP thin film, a polymeric phase is selectively infiltrated with a metal-containing precursor and then exposed to an oxygen-based agent, such as H_2_O, to generate metal oxides inside the polymeric matrix. After infiltration, the removal of the polymer scaffold by O_2_ plasma yields inorganic nanostructures mimicking the selected polymer domain. SIS in BCPs could guarantee the fabrication of a wide variety of mixed organic/inorganic or inorganic nanostructures because of the reaction of the precursors within one phase of the self-assembled BCP.^13,20,40,47,48^ Stripes and dots have been obtained by using perpendicularly oriented lamellae and cylinder-forming BCP thin films as templates.^31,36,49,50^ Poly(styrene)-*block*-poly(methyl methacrylate) (PS-*b*-PMMA) BCPs have been selected as the favorite material for lithographic applications.^51^ The capability to achieve perpendicularly oriented nanodomains is basic for their exploitation as templates for nanofabrication processes. Among the different approaches, the deposition of homopolymer or random copolymer (RCP) thin films has been tested as the base option to balance the interfacial interactions between the BCPs and the surface.^52−54^ On the other hand, gyroid structures can be directly exploited for optical applications.^55^

The fine-tuning of the dimensions of the inorganic nanostructures can be achieved by proper combination of the SIS process conditions. In particular, the sequential reaction steps of the SIS process allow tuning the dimensions of the inorganic nanostructures modifying the number of SIS cycles.^56^ This capability to finely tune the dimensions of the resulting inorganic nanosctructures is essential to make this technology suitable for the different applications. At the same time, this incremental growth of the inorganic nanostructures allows delving into the growth mechanism monitoring the progressive mass uptake of the inorganic component into the polymer scaffold.^57^

**Figure 1:**
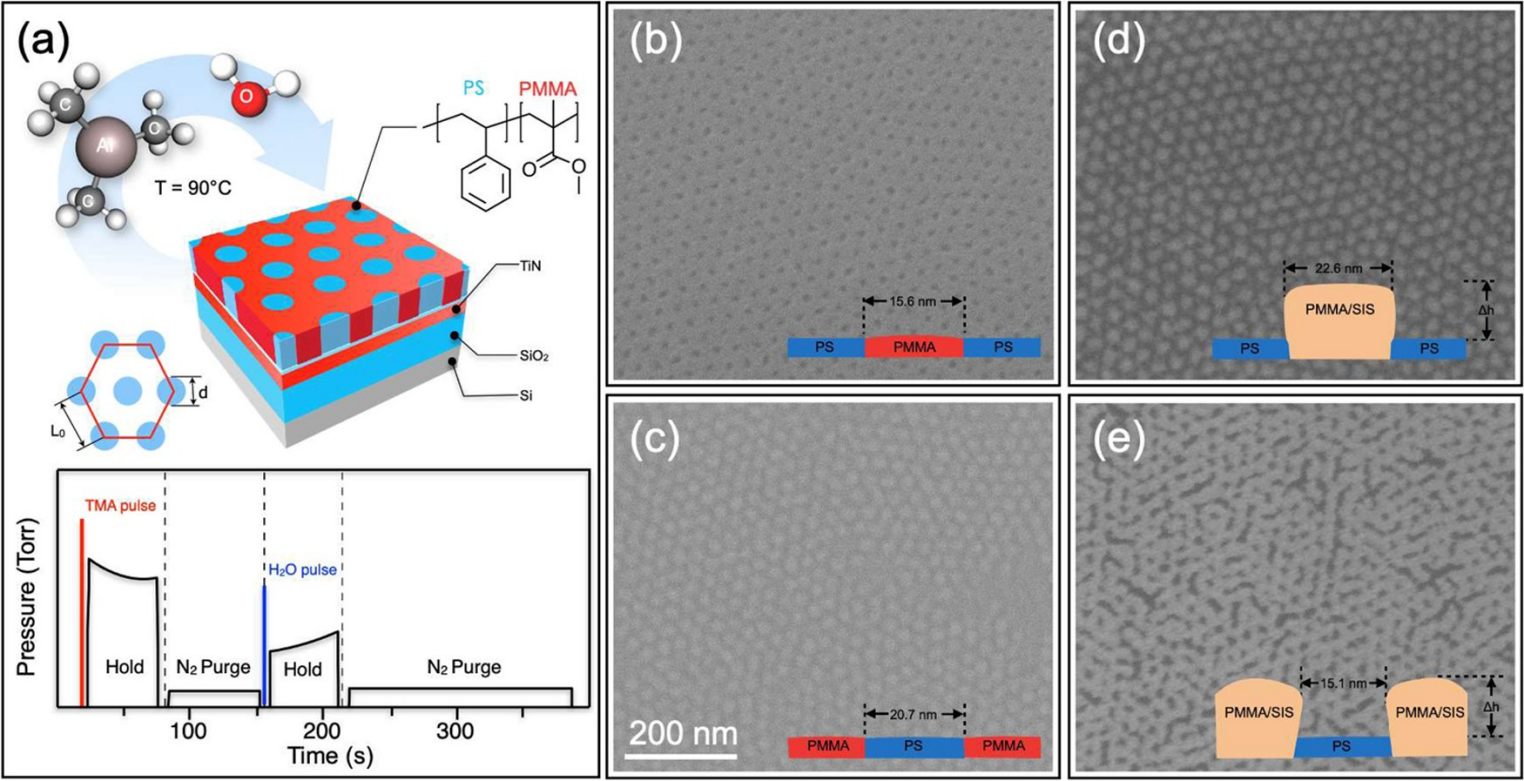
Scheme depicting the experimental processing conditions for infiltration of Al_2_O_3_ into self-assembled cylinder-forming PS-*b*-PMMA thin films (a). Plan-view SEM images of the original BCP templates with out-of-plane hexagonally packed PMMA cylinders embedded in a PS matrix (b) and with out-of-plane hexagonally packed PS cylinders embedded in a PMMA matrix (c). Plan-view SEM images of the same BCP templates upon infiltration of Al_2_O_3_ into the PMMA phase by four SIS cycles (d,e). Insets provide a schematic representation of the swelling induced by Al_2_O_3_ incorporation into the PMMA phase of the BCP templates.

As highlighted by Peng et al.,^56^ the SIS approach can be considered as a controllable molecular assembly process where the polymer chains in phase-separated BCP domains are used as a molecular frame for templating material growth. The basic cycle sequence is composed of two half cycles, one for the metal precursor and one for the oxygen precursor. The sequence of each half cycle is similar. It is composed of a pulse of the metal or oxygen precursor in the chamber followed by an exposure step to promote incorporation of the precursor molecules into the polymer matrix. Then, a purge of the chamber by ultrapure N_2_ is performed to remove unreacted precursor molecules from the chamber atmosphere. A simple scheme of this pulse/exposure/purge (PEP)^58^ sequence is depicted in Figure 1a. Leng and Losego^4^ highlighted that the fundamental physical processes involved in the SIS growth are sorption and diffusion of the precursor molecules into the polymeric matrix and reaction of these molecules within the polymer matrix.^6,7,11,12,59^ Each of these processes affects the characteristics of the resulting nanostructures.^35,60^ To get a clearer picture of the fundamental mechanisms of SIS growth, a better understanding of the role of these processes as well as of their interplay is required.

In this work, two perpendicularly oriented cylinder-forming PS-*b*-PMMA BCP thin films with different “specular” PMMA volume fractions were infiltrated iterating the number of SIS cycles and keeping fixed the PEP sequence. Cylinder-forming PS-*b*-PMMA BCP thin films having PMMA as a minor (major) component were considered to generate polymeric ∼40 nm-thick films with out-of-plane hexagonally packed PMMA (PS) cylinders having periodicity *L*_0_ = 35.5 nm (31.5 nm). These BCP thin films were infiltrated with trimethylaluminum (TMA) and H_2_O at 90 °C in a crossflow reactor operating in quasi-static mode to incorporate Al_2_O_3_ into the PMMA matrix. Upon clearing of the polymer matrix by O_2_ plasma treatment, the resulting hexagonally organized Al_2_O_3_ dot and antidot arrays were characterized by means of scanning electron microscopy (SEM) and ex situ spectroscopic ellipsometry (SE), providing information about the characteristic dimensions of these nanostructures, that is, Al_2_O_3_ dot (antidot) diameter and height (thickness) as a function of the number of SIS cycles. BCP-based lithography has been investigated as an option for advanced lithographic applications. Al_2_O_3_ dot and antidot arrays are particularly relevant because of their exploitation as hard masks with higher selectivity for the subsequent additive or subtractive steps in the process flow of the semiconductor device fabrication.^20−25^ Based on these experimental data, the overall mass uptake in the different BCP systems was estimated and compared with mass uptake in PMMA thin films that were used as references. Collected experimental data shed new light on the effect of the nanostructured polymeric template on the effective introduction of Al_2_O_3_ into the PMMA matrix during the SIS process. We can anticipate that mass uptake of Al_2_O_3_ into the PMMA component of PS-*b*-PMMA is higher than that in PMMA thin films.

## Results

Approximately 40 nm-thick PS-*b*-PMMA BCP thin films with PMMA volume fractions *f*_MMA_ ∼ 0.3 and *f*_MMA_ ∼ 0.7 were deposited onto Si wafers bearing a TiN/SiO_2_ stack. Prior to BCP deposition, the TiN surface was neutralized by a RCP brush layer to prevent preferential wetting phenomena and promote out-of-plane orientation of nanodomains in the self-assembled BCP thin films. Figure 1a shows a scheme of the sample structure and of the SIS processing conditions. The morphology of the self-assembled PS-*b*-PMMA thin films has been investigated by SEM. Representative plan-view SEM images of the PS-*b*-PMMA asymmetric BCP templates for the matrix with hexagonally packed PMMA cylinders (dark gray) inside the PS matrix (light gray) and for the matrix with hexagonally packed PS cylinders (light gray) inside the PMMA matrix (dark gray) are reported in Figure 1b,c, respectively. From software analysis of the SEM images, the diameter of the PMMA cylinders was evaluated to be *d* = 15.6 ± 0.3 nm and their center-to-center distance *L*_0_ = 35.5 ± 0.5 nm. Similarly, the diameter and the center-to-center distance of the PS cylinder were determined to be *d* = 20.7 ± 0.5 nm and *L*_0_ = 31.5 ± 0.4 nm, respectively.

Figure 1d,e shows representative SEM plan-view images of the same samples upon four SIS cycles at 90 °C using TMA and H_2_O as metal and oxygen precursors, respectively. Incorporation of Al_2_O_3_ into the BCP templates results in the formation of hybrid organic–inorganic structures and determines a significant swelling of the PMMA phase for the infiltrated PMMA cylinders and the infiltrated PMMA matrix, according to the schemes that are reported in the insets of Figure 1d,e, respectively. The average diameter of the infiltrated PMMA cylinders has been increased to *d* = 22.7 ± 0.7 nm. Conversely, because of the swelling of the PMMA phase the average diameter of the PS cylinders has been reduced to *d* = 15.1 ± 0.7 nm. In both cases, the hexagonal patterns have been preserved, and *L*_0_ is unaffected by the SIS process. These experimental results are perfectly consistent with atomic force microscopy (AFM) investigation reported by Lorenzoni et al.^11^

**Figure 2:**
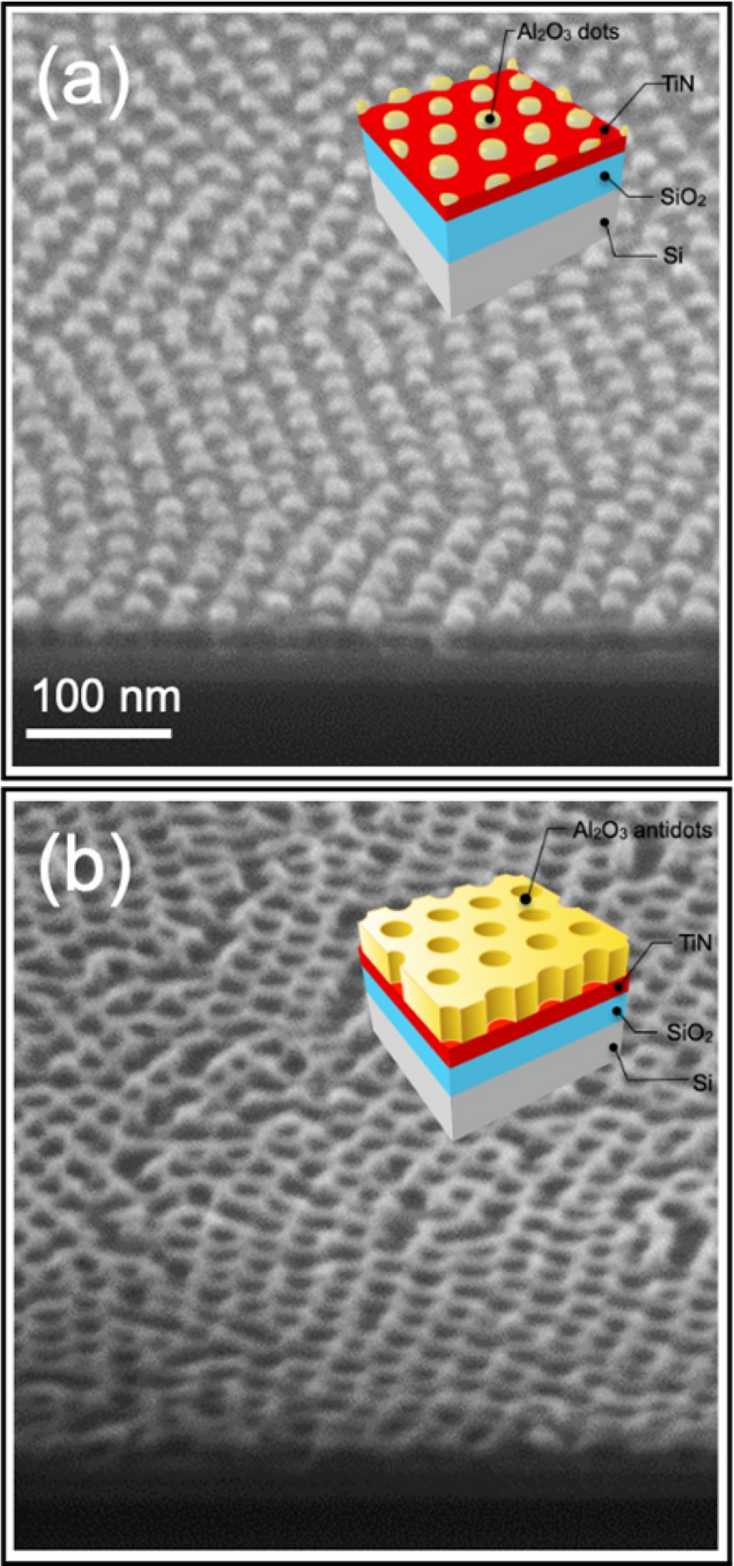
30° tilted SEM images of the Al_2_O_3_ dot (a) and antidot (b) arrays upon four SIS cycles and subsequent removal of the polymeric phase by O_2_ plasma treatment. Insets provide a schematic representation of the final structure of the samples.

Upon clearing of the polymer matrix by O_2_ plasma treatment and the concomitant aggregation of Al_2_O_3_ nuclei on the underlying substrate, Al_2_O_3_ nanostructures were formed on top of the TiN surface, as shown in the tilted SEM images of Figure 2a,b, respectively. These representative images evidence that the Al_2_O_3_ structures mimic the morphology of the PMMA component in the original BCP templates. The final structures of the samples upon removal of the organic phase are schematically depicted in the insets of Figure 2a,b. In particular, the inorganic nanostructures obtained from the BCP thin films with PMMA cylinders embedded in a PS matrix are hexagonally packed Al_2_O_3_ dot arrays. Conversely, in the case of BCP thin films with PS cylinders in a PMMA matrix, hexagonally packed Al_2_O_3_ antidot arrays are formed.^40^ In both cases, the hexagonal morphology of the original BCP template was perfectly preserved both in the hybrid organic–inorganic templates obtained by the infiltration process and in the final inorganic structures obtained by removal of the organic phase upon O_2_ plasma treatments.^36,56^

**Figure 3:**
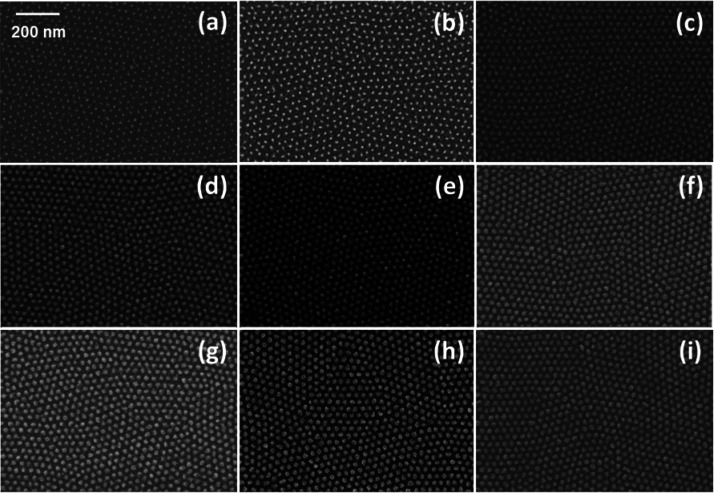
Collection of plan-view SEM images of the Al_2_O_3_ dot arrays obtained by infiltration of TMA and H_2_O into BCP thin films with out-of-plane hexagonally packed PMMA cylinders embedded in a PS matrix and subsequent removal of the polymeric phase by O_2_ plasma treatment. Al_2_O_3_ dot arrays were prepared with 1 (a), 2 (b), 3 (c), 4 (d), 5 (e), 6 (f), 7 (g), 9 (h), and 11 (i) SIS cycles.

The sequential upload of Al_2_O_3_ during the SIS process allowed controlling the dimension of the Al_2_O_3_ structures by increasing the number of SIS cycles, keeping fixed all the other process parameters. Figure 3 reports plan-view SEM images of the Al_2_O_3_ dot arrays obtained infiltrating the BCP template composed of hexagonally packed PMMA cylinders embedded in a PS matrix with different numbers of SIS cycles. Interestingly, a single SIS cycle is already enough to obtain an Al_2_O_3_ morphology that mimics the PMMA component of the BCP template with almost no defects. Increasing the number of SIS cycles, the average diameter of the Al_2_O_3_ dots increases maintaining fixed *L*_0_, that is dictated by the BCP properties.

**Figure 4:**
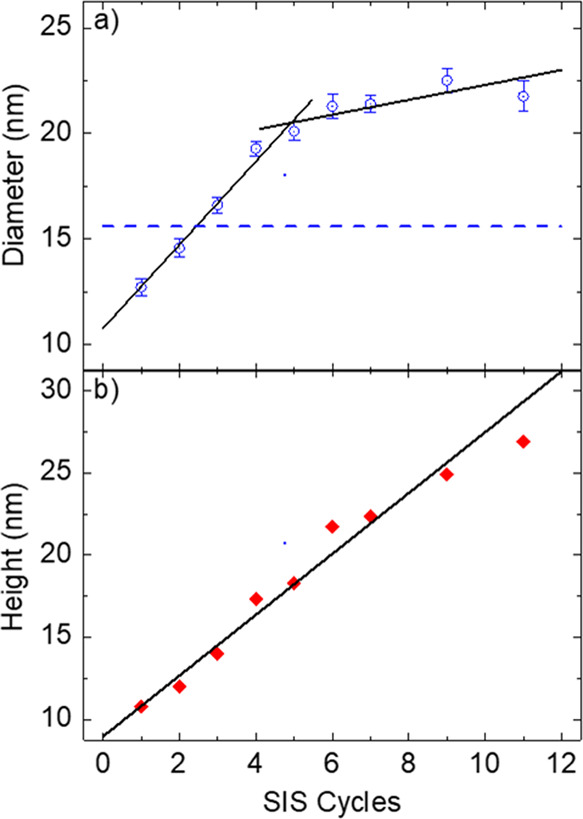
(a) Diameter of the Al_2_O_3_ dots obtained from software analysis of SEM plan-view images and (b) height of the Al_2_O_3_ dots obtained by fitting of SE data are reported as a function of the number of SIS cycles. The blue dashed line indicates the average diameter of the PMMA cylinders in the original phase-separated BCP thin film. Black lines correspond to linear fits of the experimental data.

Through software analysis of the SEM plan-view images, the evolution of the diameter of the Al_2_O_3_ dots was investigated as a function of the number of SIS cycles (Figure 4a). The blue dashed line indicates the average diameter (*d* = 15.6 nm) of the PMMA cylinders in the original phase-separated BCP thin film, as measured from the plan-view SEM image reported in Figure 1a. The diameter of the Al_2_O_3_ dots overcomes that of the original PMMA cylinders after three SIS cycles, increasing steeply at first and then more gradually as a function of the number of SIS cycles, with a well-defined threshold at five SIS cycles. These two growth regimes are characterized by a linear increase of the dot diameter. By linear fitting of the experimental data (black solid lines), the slopes in the two regimes were found to be 2.0 and 0.35 nm/cycle, respectively. In addition, the evolution of the height of these nanostructures was investigated by SE measurements. Ellipsometry data were fitted using a Cauchy model to describe the Al_2_O_3_ dot arrays. As shown in Figure 4b, the height of the Al_2_O_3_ dots monotonically increases with a single linear regime having a slope of 1.85 nm/cycle (black solid line). The height of the Al_2_O_3_ dots is always smaller than the thickness of the original BCP template. AFM measurements were performed to countercheck the SE measurement for selected samples. AFM data were found to be in excellent agreement with the height values obtained by SE analysis (S1–S5).

**Figure 5:**
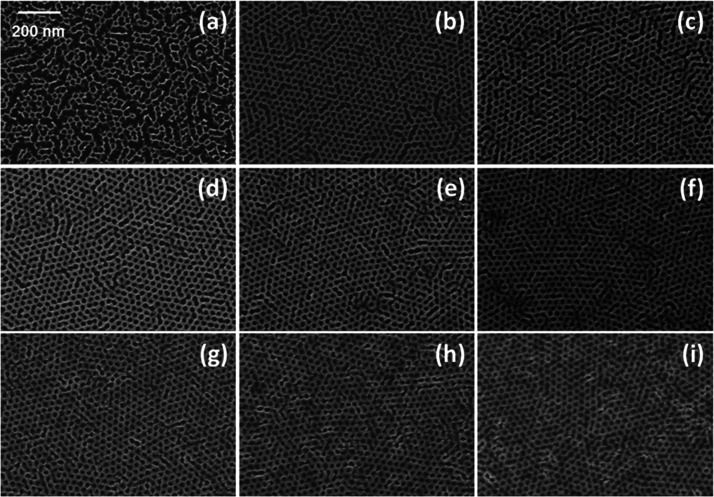
Collection of plan-view SEM images of the Al_2_O_3_ antidot arrays obtained by infiltration of TMA and H_2_O into BCP thin films with out-of-plane hexagonally packed PS cylinders embedded in a PMMA matrix and subsequent removal of the polymeric phase by O_2_ plasma treatment. Al_2_O_3_ antidot arrays were prepared with 1 (a), 2 (b), 3 (c), 4 (d), 6 (e), 8 (f), 10 (g), 12 (h), and 15 (i) SIS cycles.

Figure 5 reports the plan-view SEM images of hexagonally packed Al_2_O_3_ antidot arrays obtained infiltrating the BCP thin films with a PMMA matrix surrounding hexagonally packed PS cylinders, with different numbers of SIS cycles. It is worth noticing that, in the case of the Al_2_O_3_ antidot array obtained with a single SIS cycle, the inorganic matrix is not continuous, while increasing the number of SIS cycles a continuous Al_2_O_3_ antidot array is formed. In the original BCP template, the PMMA component occupies about 70% of the polymer matrix. This large PMMA volume could partially justify the inability to fill all the PMMA components with a single SIS cycle. A similar effect was reported by Xiong et al. in the case of P2VP upon one SIS cycle using the same precursors.^61^ Correspondingly, Ruiz et al. observed a similar phenomenon in the case of infiltration of a PMMA matrix in a lamellar BCP with TMA and H_2_O.^25^

**Figure 6:**
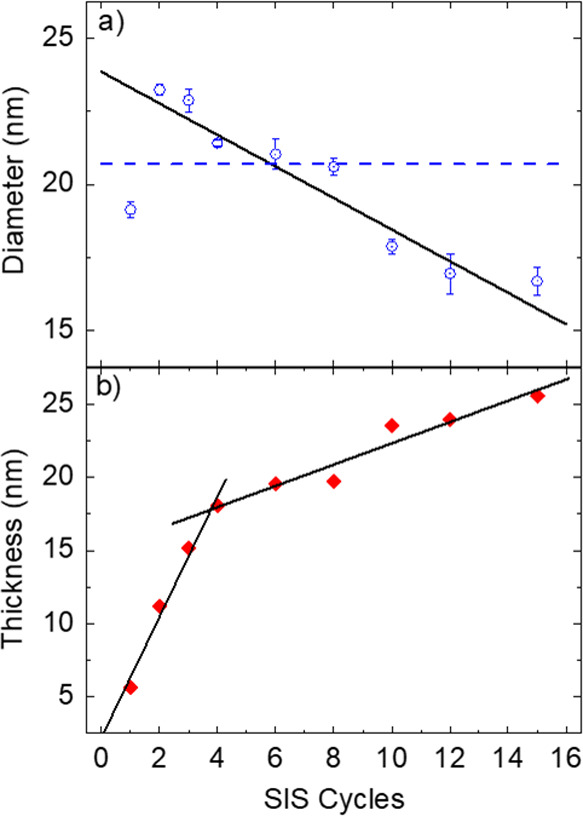
(a) Diameter of the holes in the Al_2_O_3_ antidot arrays obtained by software analysis of the SEM images and (b) thickness of the Al_2_O_3_ antidot arrays obtained by fitting of the SE data as a function of the number of SIS cycles. The blue dashed line indicates the average diameter of the PS cylinders in the original phase-separated BCP thin film. Black lines correspond to linear fits of the experimental data.

Figure 6a reports the evolution of the hole diameter in the Al_2_O_3_ antidots arrays as a function of number of the SIS cycles, as measured from software analysis of plan-view SEM images. In this case, except the sample infiltrated with a single SIS cycle where the structure is not continuous, the average diameter of the holes in the Al_2_O_3_ antidot arrays linearly decreases when increasing the number of SIS cycles. By linear fitting (black solid line) of the experimental data, an almost constant decrease of −0.55 nm/cycle is measured. The dashed line in Figure 6a indicates the diameter (*d* = 20.7 nm) of the PS cylinders in the initial polymer template. Upon six SIS cycles, the average diameter of the holes in the Al_2_O_3_ antidot arrays becomes smaller than the diameter of the PS cylinders (blue dashed line) in the initial polymer template. The thickness of the Al_2_O_3_ antidot arrays was monitored by SE. Figure 6b shows the evolution of the thickness of the Al_2_O_3_ antidot arrays as a function of the number of SIS cycles. According to the collected data, the Al_2_O_3_ antidot arrays are always thinner than the original BCP thin film irrespective of the number of SIS cycles. Moreover, the thickness of the Al_2_O_3_ antidot array monotonically increases, and two different regimes are clearly observed. Black solid lines in Figure 6b correspond to linear fittings of the experimental data. During the initial stages of the SIS process, a fast linear increase of 4.1 nm/cycle is observed. Upon four SIS cycles, the thickness increase slows down, still following a linear evolution but at a much lower rate of 0.7 nm/cycle.

**Figure 7:**
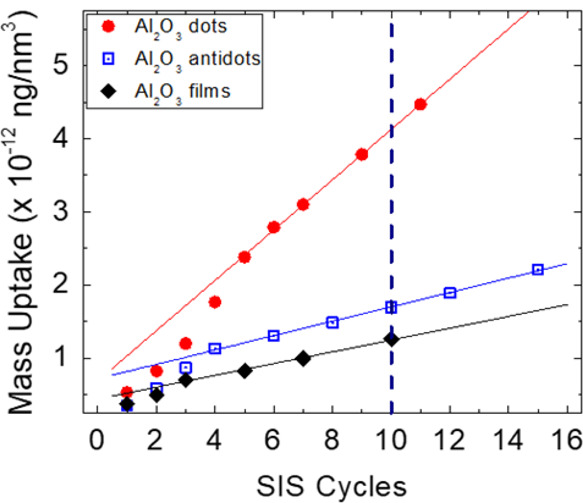
Mass uptake in out-of-plane hexagonally packed PMMA cylinders, PMMA cylinders embedded in a PS matrix, out-of-plane hexagonally packed PS cylinders embedded in a PMMA matrix, and continuous PMMA film as resulting Al_2_O_3_ mass over the available PMMA volume in the initial polymeric matrix as a function of the number of SIS cycles. Solid lines correspond to linear fitting of the experimental data.

Based on the collected data, the overall mass uptake of Al_2_O_3_ with respect to the polymeric content of PMMA in the BCP template was evaluated. The calculation was performed modeling the Al_2_O_3_ dot array as a matrix of perfect Al_2_O_3_ cylinders having diameter corresponding to the one measured from software analysis of the SEM images and height corresponding to the one obtained from SE. Similarly, the Al_2_O_3_ antidot array was modeled as a mesoporous film with thickness equivalent to the one obtained from SE analysis and holes propagating throughout the entire film thickness. The pore diameter was assumed to correspond to the one obtained from analysis of plan-view SEM images. Finally, we assume a constant density for Al_2_O_3_ of 2.70 × 10^–12^ ng/nm^3^ for all the nanostructures, consistently with data reported in the literature.^62^Figure 7 reports the calculated mass uptakes for the two different morphologies. These data are compared with those obtained in the case of infiltration of TMA and H_2_O in a continuous PMMA film having thickness *h* ∼ 45 nm. Interestingly, all the samples exhibit similar evolutions as a function of the number of SIS cycles: after an initial regime characterized by a fast incorporation of Al_2_O_3_, the mass uptake increases linearly, indicating that in this regime a constant amount of Al_2_O_3_ is incorporated in a fixed volume of PMMA at each SIS cycle. In this regime, the growth rate, that is, the mass uptake at each cycle, can be calculated from the linear fitting of the data reported in Figure 7. Accordingly, the average growth rate at each cycle of the SIS process is determined to be 34.4 × 10^–14^, 9.8 × 10^–14^, and 8.1 × 10^–14^ (ng/nm^3^) for the Al_2_O_3_ dot array, for the Al_2_O_3_ antidot array, and for the continuous Al_2_O_3_ film, respectively. These data indicate that, in polymer films having approximately the same thickness, the PMMA phase in the self-assembled BCP templates incorporates a larger amount of Al_2_O_3_ per unit volume of PMMA, than the PMMA in a continuous film. Moreover, among the nanostructured PMMA, the BCP thin film with PMMA as a minor component incorporates more Al_2_O_3_ than the BCP thin film with PMMA as a major component. In the latter case, the mass uptake is similar to the one observed in the case of the continuous PMMA film, suggesting that this BCP morphology can be qualitatively modeled as a continuous PMMA film with a reduced volume due to the presence of the hexagonally packed PS cylinders.

**Figure 8:**
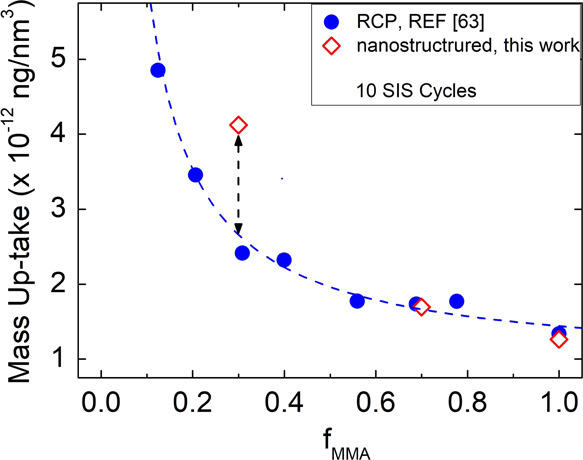
Mass uptake data upon 10 SIS cycles for nanostructured BCP templates with different PMMA contents and for a continuous PMMA film (red open symbols) are compared with mass uptake results for RCP thin films (blue solid symbols). Data are reported as a function of volume fraction of MMA (*f*_MMA_) in the polymer matrix. Mass uptake data for RCP thin films are obtained by ref (63).

To further clarify these experimental results, collected data are compared with those obtained in P(S-*r*-MMA) RCP thin films with similar thickness *h* ∼ 55 nm and various MMA volume fractions. In a recent paper, Caligiore et al. demonstrated that the thickness of the Al_2_O_3_ film that is obtained upon infiltration of Al_2_O_3_ into P(S-*r*-MMA) thin films and subsequent removal of the polymer matrix by O_2_ plasma treatment increases linearly with the MMA volume fraction, demonstrating that the incorporation of Al_2_O_3_ into the polymer film is directly related to the concentration of reactive sites in the polymer matrix. Moreover, the same paper evidenced that the diffusion of TMA is fast enough to infiltrate the whole volume of the 55 nm-thick P(S-*r*-MMA) and PMMA films. Accordingly, the amount of Al_2_O_3_ grown into the polymeric film during the SIS process was considered to be essentially limited by the number of reactive sites in the system.^63^ In Figure 8, these literature data (blue closed circles) are reported in terms of mass uptake upon 10 SIS cycles. Mass uptake was calculated as the amount of Al_2_O_3_ that is incorporated into the polymeric film per unit volume of PMMA. According to the previously described protocol, calculation was performed assuming a constant density for Al_2_O_3_. To facilitate data comparison, in Figure 8 mass uptake values (red open squares) obtained upon 10 SIS cycles in the self-assembled BCP thin films are reported as a function of the volume fraction of PMMA in the original BCP template. We can observe that with decreasing the PMMA fraction, mass uptake increases both for the RCP and the self-assembled BCP films. For the PS-*b*-PMMA thin films with *f*_MMA_ ∼ 0.7, mass uptake is almost equivalent to the one of the RCP thin films having the same MMA volume fraction. Differently, the mass uptake for the PS-*b*-PMMA thin films with *f*_MMA_ ∼ 0.3 is much higher than the one of the RCP thin films with the same MMA volume fraction. From a general point of view, these data indicate that, when the nonreactive PS component in the copolymer system increases, the capability of the reactive PMMA component to incorporate Al_2_O_3_ increases. This effect is even more pronounced in the case of self-assembled PS-*b*-PMMA templates where the two components are phase-separated and organized in well precise morphologies.

**Figure 9:**
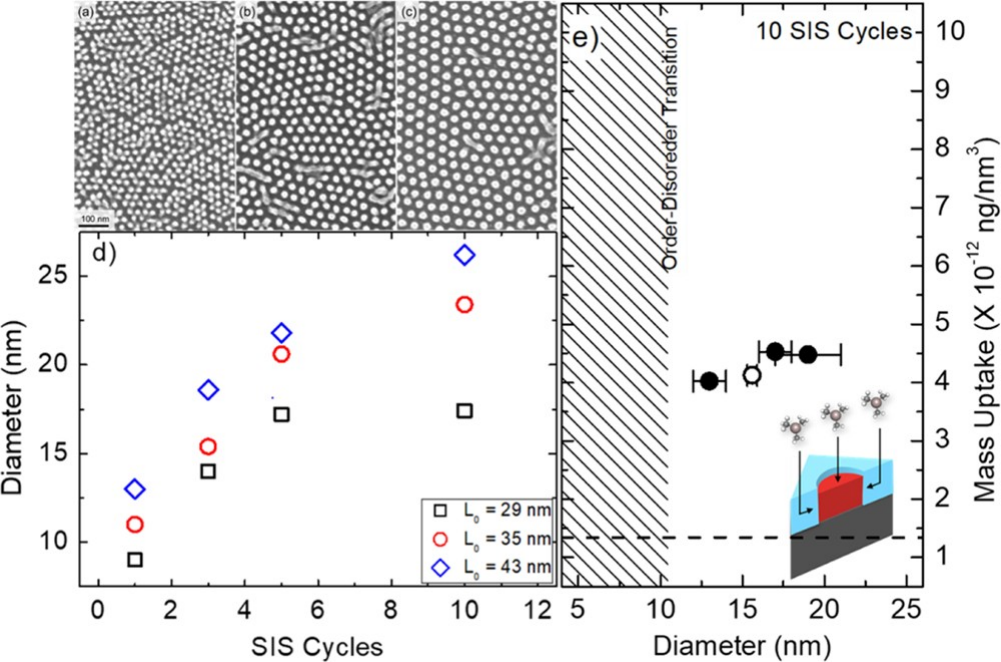
Collection of plan-view SEM images for the Al_2_O_3_ dot array obtained from self-assembled PS-*b*-PMMA thin films with the diameter of PMMA cylinders equivalent to 13 nm (a), 17 nm (b), and 19 nm (c) upon infiltration with 10 SIS cycles at 90 °C and subsequent removal of the polymer phase by O_2_ plasma treatment. (d) Diameter of the Al_2_O_3_ dots as a function of the number of SIS cycles for PS-*b*-PMMA thin films with different diameters and *L*_0_ (29, 35, and 43 nm, respectively) of the PMMA cylinders. (e) Mass uptake after 10 SIS cycles for PS-*b*-PMMA thin films as a function of diameters of the PMMA cylinders. The black dashed line represents the mass uptake in a homogeneous PMMA film with similar thickness that was used as a reference.

To further investigate this effect, we studied the infiltration process in three different PS-*b*-PMMA having the same MMA volume fraction *f*_MMA_ ∼ 0.3 but different molecular weight (*M*_n_) equal to 54, 67, and 82 kg/mol, respectively. Upon annealing, these BCPs self-assemble in hexagonally packed PMMA cylinders embedded in a PS matrix. The diameter of the PMMA cylinders is *d* = 13.0 ± 1.0, 17 ± 1.0, and 19 ± 2.0 nm for the BCP with *M*_n_ equal to 54, 67, and 82 kg/mol, respectively. The BCP templates were infiltrated using the same SIS process that was used in the previously reported systematic study for the formation of Al_2_O_3_ dot and antidot arrays. Upon infiltration and removal of the polymer template by O_2_ plasma, hexagonally packed Al_2_O_3_ dot arrays were obtained. Representative plan-view SEM images for the Al_2_O_3_ dot array obtained from self-assembled PS-*b*-PMMA thin films with diameter of PMMA cylinders equivalent to 13, 17, and 19 nm and *L*_0_ equivalent to 29, 35, and 43 nm, respectively, upon infiltration with 10 SIS cycles at 90 °C and subsequent removal of the polymer phase by O_2_ plasma treatment are reported in Figure 9a–c. SEM analysis highlighted that the average diameter of the Al_2_O_3_ dots progressively increases with the number of SIS cycles. Figure 9d reports the evolution of the diameter of the Al_2_O_3_ dots as a function of the number of SIS cycles for this set of BCP templates as measured from software analysis of plan-view SEM images. The evolution of the diameters as a function of the SIS cycles follows the same trend already observed for this specific BCP system in Figure 4a. The diameters shift proportionally to the *M*_n_ of the specific BCP that was used to generate the nanostructured polymeric template. Based on these data, following the same protocol that was previously discussed, we calculated the mass uptake for the hexagonally packed Al_2_O_3_ dot arrays upon 10 SIS cycles. Figure 9e reports the mass uptake values (closed symbols) as a function of the diameter of the PMMA cylinders in the self-assembled PS-*b*-PMMA thin film for this set of samples. The mass uptake value (open symbol) that was previously calculated based on data reported in Figure 7 for PS-*b*-PMMA BCP thin films with PMMA cylinders having diameter *d* = 15.6 nm is reported as well. The black dashed line represents the mass uptake in a homogeneous PMMA film with a similar thickness that was used as a reference. According to these data, mass uptake is independent of the diameter of the PMMA cylinders in the original BCP template within the investigated range.

## Discussion

From a fundamental point of view, collected data provide interesting information about the mechanism governing the incorporation of Al_2_O_3_ into the PMMA matrix. The most important result is that, assuming the same processing conditions during the SIS process, mass uptake into self-organized PS-*b*-PMMA thin films is larger than in a homogeneous PMMA film, in agreement with data reported in the literature. In a seminal paper about the infiltration of TiCl_4_ and H_2_O precursors in cylinder-forming PS-*b*-PMMA thin films, Peng et al. compared the growth of TiO_2_ in the self-assembled BCP thin films and in a continuous PMMA film. The PS phase was identified as the main channel to deliver TiCl_4_ molecules to the PMMA phase. Additionally, the interface between PS and PMMA was shown to provide reactive sites for SIS reaction and the PMMA domains were shown to exhibit a higher TiCl_4_ diffusion rate and higher desorption rate than a continuous PMMA film. Overall, these features implied that the trapping of the metal precursor within PMMA nanodomains is more efficient that in a homogenous PMMA film due to the presence of the percolation pathways provided by the PS domains inside the self-assembled BCP film.^56^ The fundamental role of the interface between the polymeric domains was investigated using a different approach by Berman and Shevchenko.^15,27,29^ They took advantage of the swelling of the PS-*b*-P4VP matrix to control the porosity of BCP materials. BCP swelling increases free volume in the polymer matrix and consequently diffusivity of TMA and H_2_O precursors promoting incorporation of Al_2_O_3_ into the P4VP component of the polymer matrix. These studies are in perfect agreement with data herein reported and suggest that the nonreactive PS component plays a fundamental role in promoting incorporation into the reactive PMMA nanodomains. In particular, the PS component provides additional diffusion paths that promote the incorporation of Al_2_O_3_ into the reactive PMMA component of the polymer films. This effect is even more evident in the case of nanostructured BCP thin films with hexagonally packed PMMA cylinders embedded into a PS matrix, where the nonreactive PS component surrounds the reactive PMMA component. As schematically depicted in the inset of Figure 9, this specific morphology guarantees the possibility to infiltrate Al_2_O_3_ into the PMMA volume following different diffusion paths: a first path is related to sorption of TMA and H_2_O precursors at the top surface of the PMMA cylinders with subsequent diffusion through the PMMA matrix, while a second path is associated to diffusion of TMA and H_2_O precursors through the nonreactive PS matrix and subsequent sorption at the lateral surface of the PMMA cylinders, that is, at the PS/PMMA interface. In this respect, it is necessary to better clarify the role of the nonreactive PS component during the SIS process. It is widely reported that Al_2_O_3_ preferentially grows in the PMMA component of self-assembled BCP films. However, Cianci et al. pointed out that defects in the PS films can act as reaction sites for TMA molecules. In particular, the growth of Al_2_O_3_ inside the PS phase is possible upon several SIS cycles when defects are present as nucleation sites for the growth.^58^ Similarly, Peng et al. noted a decrease in selectivity between PMMA and PS upon six SIS cycles when the rough material start to be incorporated into the PS domains. This feature was ascribed to the nucleation of Al_2_O_3_ on the PS domains due to physical trapping of reactants because of the inert chemistry of PS. The latter effect was reported to increase when increasing the number of cycles.^56^ In the present work, no evidence of Al_2_O_3_ incorporation into the PS matrix is observed in the nanostructured BCP thin films irrespective of their morphology. Cianci et al. demonstrated that TMA diffusivity in PMMA and PS is roughly the same. Considering the thickness of the BCP thin films that were used in this experiment, TMA is expected to diffuse through the entire polymer film during the exposure step, ruling out the hypothesis that the enhancement of Al_2_O_3_ incorporation into the nanostructured BCP thin films is related to a faster diffusion of TMA in the PS matrix. Data in Figure 9 indicate that the mass uptake is almost independent of the diameter of the PMMA cylinder, indicating that, for this range of values, mass uptake does not scale with the area of the PMMA/PS interface. Accordingly, this result suggests that, in this specific configuration, the incorporation of Al_2_O_3_ into the PMMA cylinders is not limited neither by the sorption nor by the diffusion and the additional diffusion path provided by the PS matrix is extremely effective to deliver the TMA precursor to the PMMA phase increasing the mass uptake with respect to homogeneous PMMA films. It is worth noting that, according to previous studies, in these specific systems diffusivity is high enough to guarantee infiltration of TMA into the entire volume of the polymer film, irrespective of the film composition.^63^ Accordingly, the enhancement of mass uptake in the nanostructured PS-*b*-PMMA with respect to P(S-*r*-MMA) with the same styrene fraction could be tentatively associated with an enhancement of TMA sorption at the PMMA/PS interface.

From a technological point of view, collected data indicate that the fabrication of inorganic nanostructures by means of SIS in BCP guarantees an accurate tuning of the final dimensions of the inorganic nanostructures by properly controlling the process parameters. For the Al_2_O_3_ antidot arrays, Zhou et al. showed a linear dependence of the pore size on the number of the SIS cycles, demonstrating that this behavior holds up to five SIS cycles. In this limited range, the decrease of the pore size was reported to be around 25% when increasing the number of SIS cycles from 1 to 5.^36^ In our system, the decrease of the pore size appears to be slower. Nevertheless, it is worth noticing that the SIS processes that were used in the two cases are characterized by PEP subcycles for the two precursors that are very different. In a previous experiment, Cianci et al. demonstrated that a change of the PEP sequence implies a significant variation in the amount of Al_2_O_3_ that is incorporated in a PMMA matrix. Moreover, the thickness of the BCP templates is different in the two experiments. By further increasing the number of SIS cycles, an additional decrease of the pore size is observed, demonstrating the capability to finely tune the average diameter of the pores, approaching the 15 nm limit. At the same time, the thickness of the Al_2_O_3_ antidot array is observed to increase as a function of the number of SIS cycles with two different growth regimes: an initial regime characterized by a rapid evolution of the thickness followed by a second regime exhibiting a significant decrease of the growth rate. A similar thickness evolution was already reported by Cianci et al. for PMMA films of thickness ranging from 8 to 100 nm.^58^ In that study, TMA molecules were demonstrated to diffuse throughout the entire film during the SIS process for the specific range of polymer thickness values under consideration, ruling out the hypothesis that mass uptake is limited by the thickness of the polymer film.

As already highlighted, in the Al_2_O_3_ dot arrays, the height and average diameter of the Al_2_O_3_ dots follow similar but opposite trends. The height of the Al_2_O_3_ dots linearly increases as a function of the number of SIS cycles. Conversely, a rapid evolution of their average diameter is observed during the early SIS cycles followed by a second regime exhibiting a significant decrease of the growth rate. Data reported in Figure 9 evidence the possibility to obtain Al_2_O_3_ dots with the average diameter below 15 nm. In principle, further reduction of the diameter of the Al_2_O_3_ dots could be possible by considering PS-*b*-PMMA with smaller molecular weight. However, the molecular weights of the PS-*b*-PMMA that were considered in this experiment are quite close to the minimum value that allows achieving efficient phase separation of PS-*b*-PMMA suggesting that the values herein reported are quite close to the limit for this specific BCP system.^64^ For these specific BCPs, the density of dots/inch^2^ ranges from ∼0.9 to 0.4 × 10^12^ dots/inch^2^ for *M*_n_ values ranging from 54 to 82 kg/mol, respectively.^65^ Finally, it is worth noting that according to these data by proper selection of the molecular weight of the PS-*b*-PMMA and of the number of SIS cycles it is possible to fabricate Al_2_O_3_ dot arrays with the same average diameter of the Al_2_O_3_ dots but different pitches, providing the capability to independently control on pitch and diameter in the Al_2_O_3_ dot arrays.

In wider terms, antidot arrays can be compared with ordered mesoporous alumina (OMA).^66^ OMA can be fabricated by the evaporation-induced self-assembly (EISA) process that is a ligand-assisted solvent evaporation-induced coassembly route.^67^ It relies on a soft template (poly(ethylene oxide)-*block*-polystyrene), a precursor (aluminum acetylacetonate), and a solvent (tetrahydrofuran). The mesostructured composites are converted into ordered mesoporous carbon-Al_2_O_3_ through pyrolysis treatment in N_2_ at high temperature. Then, calcination in air removes the carbon support. Comparing SIS in BCP and EISA, it is worth noticing that BCP self-assembly and the SIS growth process are two well-separated steps, while EISA is a coassembly strategy. Moreover, in SIS the removal of the polymeric template is performed by O_2_ plasma that leave amorphous Al_2_O_3_, while in the latter after calcination in air at 900 °C the resulting OMA shows a well-crystalline structure. On the other hand, both strategies result in mesostructures with the pore size in the range between 15 and 20 nm.

From a more applicative point of view, these Al_2_O_3_ dot and antidots arrays have characteristic dimensions and densities that are well within or even below the targets for staggered hole and pillar arrays that are investigated in process flow for semiconductor device fabrication.^68^ Testing of SIS processes on directed self-assembled BCP thin films could provide more information about the effective possibility to implement alternative lithographic approaches based on Al_2_O_3_ nanostructures to be integrated in a conventional process flow for semiconductor device fabrication.

## Conclusions

Al_2_O_3_ was infiltrated into out-of-plane cylinder-forming PS-*b*-PMMA thin films to form Al_2_O_3_ dot and antidot arrays. The evolution of the lateral and vertical dimensions of these Al_2_O_3_ nanostructures was investigated as a function of the number of the SIS cycles, operating at 90 °C and using TMA and H_2_O as metal and oxygen precursors, respectively. This systematic investigation provided information about the fundamental mechanisms steering the addition of Al_2_O_3_ into the PMMA component of the self-assembled PS-*b*-PMMA thin films: the nonreactive PS component provides additional paths for diffusion of precursor molecules into the polymer matrix significantly increasing mass uptake into the reactive PMMA component with respect to homogeneous PMMA thin films. Collected experimental data corroborate the capability of SIS to finely modify the lateral and vertical dimensions of the Al_2_O_3_ dot and antidot arrays. These hard masks are particularly well suited for the subsequent additive or subtractive steps in advanced lithographic applications. Further investigation about infiltration of Al_2_O_3_ into directed self-assembled BCP templates would be necessary to fully exploit this approach as an alternative lithographic technology and demonstrate its integrability in a conventional process flow for semiconductor device fabrication.

## Experimental Section

### Substrates

Samples with a SiO_2_/TiN stack were used. A 300 mm Si(100) substrate was cleaned followed by growth of 26 mm by thermal oxidation and deposition of a 20 nm TiN layer by physical vapor deposition. In the semiconductor industry, TiN is commonly used as a sacrificial ‘hard mask’ layer to improve back-end-of-line SiO_2_ via etching processes.

### BCP Fabrication

For the dot and the antidot matrix, the Arkema–Brewer Science OptiLign system consisting of a graftable neutral PS-*r*-PMMA layer, and a 70/30 (dots) or 30/70 (antidots) PS-*b*-PMMA block copolymer, dissolved in a propylene glycol monomethyl ether acetate (PGMEA) was used. The neutral layer was deposited by spin coating at 1500 rpm to obtain a 50 nm-thick film followed by annealing for 2 min at 220 °C on a hot plate to graft the neutral layer. The nongrafted polymers were removed by an ultrasonic rinse in PGMEA, resulting in a 7.3 nm-thick layer. Subsequently, the BCP was spin-coated at 1000 rpm to obtain an ∼36 nm (dots) or ∼40 nm (antidot) film followed by annealing for self-assembly for 5 min on a hotplate of 260 °C.^69^ Subsequently, the wafer was cleaved into samples of physical size of 1 × 1 cm.

For the dot matrix of different *L*_0_, after the cleaning process with piranha solution, a solution (18.0 mg in 2.0 mL of toluene) of a functional poly(styrene-*r*-methylmethacrylate) (P(S-*r*-MMA)) with styrene fraction 0.62 (*M*_n_ = 13.5 kg·mol^–1^ and polydispersity index (PDI) = 1.26, Polymer Source Inc.) was prepared in an ultrasonic bath and then spun on the Si(100) substrates samples of physical size of 1 × 1 cm for 30 s at 3000 rpm to obtain an ∼30 nm-thick layer. The nongrafted polymers were removed by an ultrasonic rinse in toluene, resulting in an ∼7.0 nm-thick layer.

Asymmetric PS-*b*-PMMA BCPs with different molar masses (B54, *M*_n_ = 53.8 kg/mol, *M*_n_ styrene = 37.0 kg/mol, PDI = 1.07; *L*_0_ = 29 nm, B67, *M*_n_ = 67.1 kg/mol, *M*_n_ styrene = 46.1 kg/mol, PDI 1.09; *L*_0_ = 35 nm, B82 *M*_n_ = 82.0 kg/mol, *M*_n_ styrene = 57.0 kg/mol, PDI 1.07, *L*_0_ = 43 nm) were purchased from Polymer Source Inc. and used without further purification following the already reported procedure.^65^

### SIS Process

Samples were loaded in a commercial cross flow ALD reactor (Savannah 200, Ultratech Cambridge NanoTech.) and thermalized at 90 °C for 30 min under 100 sccm N_2_ flow at 0.6 Torr before starting the infiltration. TMA and H_2_O were the metal precursor and the oxidant, respectively. Each SIS cycle consisted of successive pulses of TMA and H_2_O, each one followed by an exposure step during which the system was isolated from the pumping line, and samples were exposed to the precursor or oxidant vapor. Purging intervals under 100 sccm N_2_ flow between TMA and H_2_O pulse/exposure steps were performed. The SIS cycle was 0.025 s TMA pulse/60 s exposure/60 s purge followed by 0.015 s H_2_O pulse/60 s exposure/180 s purge. After the SIS process, the samples were washed in O_2_ plasma (40 W, 525 Torr for 10 min) that removed the polymer matrix, leaving alumina films on the Si substrate.

### Ex Situ SE

Ex situ SE was performed using a rotating compensator ellipsometer equipped with an Xe lamp (M-2000F, J. A. Woollam Co. Inc.). Ellipsometric Ψ and Δ spectra were collected over the wavelength range from 250 to 1000 nm at a fixed 75° incidence fixed angle with respect to the substrate plane normal. Spectra were modeled using the EASE software package 2.3 version (J.A. Woollam Co. Inc.), using a Cauchy layer.

### Morphological Investigation

The morphology of infiltrated polymer films upon O_2_ plasma treatment was characterized by field-emission scanning electron microscopy (FE-SEM, SUPRA 40, Zeiss) using an in-lens detector and an acceleration voltage of 15 kV. Several SEM images in different areas of each sample were acquired and analyzed using Image J.

## Supporting Information Available

AFM image of a representative area of Al_2_O_3_ dot arrays upon one, two, and three SIS cycles and a representative height profile for images upon two SIS cycles (PDF)
