# Supplementary material for: Al2O3 Dot and Antidot Array Synthesis in Hexagonally Packed Poly(styrene-block-methyl methacrylate) Nanometer-Thick Films for Nanostructure Fabrication
Source: ACS Appl Nano Mater. 2022 Jul 5;5(7):9818–28. doi: 10.1021/acsanm.2c02013 (PMC9344376; doi:10.1021/acsanm.2c02013)
Supplement: Supplementary file 1 — an2c02013_si_001.pdf [file an2c02013_si_001.pdf]

Supporting Information

Al_2O_3 Dot and Antidot Array Synthesis in Hexagonally Packed Poly(styrene-*block*-methyl methacrylate) Nanometer-Thick Films for Nanostructure Fabrication

Gabriele Seguini[1,*], Alessia Motta[1], Marco Bigatti[1], Federica E. Caligiore[1], Guido Rademaker[2], Ahmed Gharbi[2], Raluca Tiron[2], Graziella Tallarida[1], Michele Perego[1,*], and Elena Cianci[1]

[1]*IMM-CNR, Unit of Agrate Brianza, Via C. Olivetti 2, I-20864 Agrate Brianza, Italy*

[2]*Univ. Grenoble Alpes, CEA, Leti, F-38000 Grenoble, France*

*gabriele.seguini@cnr.it, *michele.perego@cnr.it

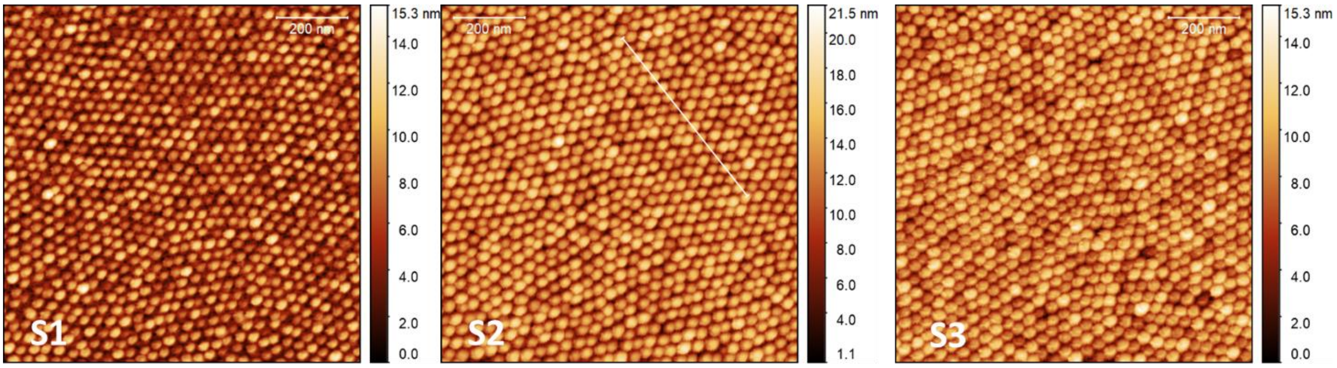

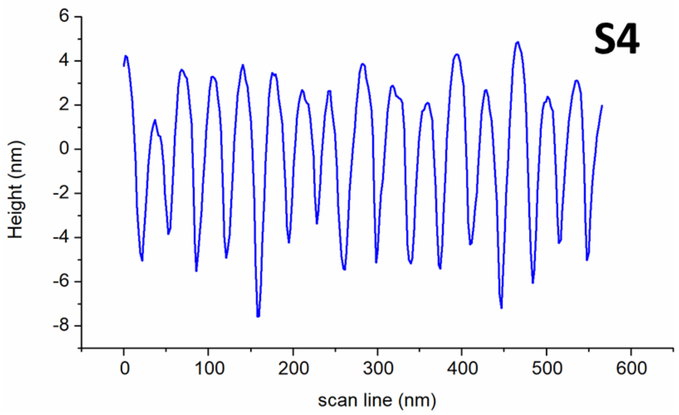

(S5)	AFM	SE
h (nm)	11.5±0.2	11.9±0.2

(**S1, S2, S3**) AFM image of a representative area of Al_2O_3 dot array upon one, two, three SIS cycles, respectively, at 90°C using TMA and H_2O as metal and oxygen precursors, respectively, in PS-*b*-PMMA asymmetric BCP templates with hexagonally packed PMMA cylinders inside the PS matrix after infiltration and upon O_2 plasma treatment.

(**S4**) representative height profile for image (S2), two SIS cycles.

(**S5**) Comparison between SE and AFM data for height values for the sample at two SIS cycles.
